# Tracheotomy—A Plea for Natural Death

**Published:** 1888-07

**Authors:** Stuart Close

**Affiliations:** Brooklyn


### TRACHEOTOMY—A PLEA FOR NATURAL DEATH.

Stuart Close, M. D., Brooklyn.

Sitting beside the deathbed of my first and only fatal case of diphtheria, watching the process of dissolution, there came to me a new realization of the beneficence of those laws of life ordained by the great Law and Life Giver, our Father.

We are accustomed to speak of natural law as "inexorable," putting into the word something of the sense of mercilessness.

It is said that "God is Love," but we too often fail to see the love as we watch these processes. Almost imperceptibly men come to have the feeling toward God that He is hard and cruel, working along certain hard and inflexible lines to accomplish His purposes, and so they gradually draw away from God, and themselves become hard and cruel. It may be that they do not become willfully or consciously so, but in the new light which came to me it seemed that they were no less truly so who rebel against natural law and its processes, and substitute for it their own will and course of action. Natural law is cruel only when disobeyed. If worked in harmony with, it is most beneficent.

Two bodies moving in parallel lines run harmoniously. It is only when the lines cross that there comes collision and disaster. This is an important truth to be learned, and is worthy of reflection.

The patient was a poor, little, deformed girl, who had been an invalid nearly all her life. Contracting diphtheria of a malignant type, she had struggled against it for six days, aided by carefully selected remedies. From the beginning, in the nature of the case, I had but little hope of her recovery.

Near the close of the sixth day, the sudden hoarseness, labored respiration, and anxious expression announced the invasion of the larynx. Remedies after this were powerless to make effectual resistance, and she sank rapidly.

For the first two hours her sufferings were painful to witness. I could scarcely refrain from trying to relieve her by performing tracheotomy. It seemed so easy a thing to do. But I remembered what I and others had seen in similar cases and withheld my hand. At the end of two hours visible signs of carbonization of the blood appeared, and *just beyond the point* where tracheotomy is usually performed, viz.: just *before* the appearance of these visible signs of carbonization, her sufferings began to abate in as marked a degree as follows, for a short time only, the operation of tracheotomy.

The poison, *created for this very purpose*, within the organism, in obedience to what I now saw for the first time to be beneficent laws, began to do its merciful work. The breathing gradually became less labored and more superficial. Consciousness, at first only dimmed, soon was lost, and an easy, almost imperceptible death followed in a few hours. This was euthanasia—a loving Father's boon to His suffering children.

After the first two hours there was little or no suffering.

Nature's narcotic was allowed full action, and it performed its merciful work well. Natural law was obeyed, and it was kind. The human will ran parallel to the Divine will, and there was no cross.

But what had I and others seen, which led me to resist my inclination to take the matter into my own hands and disregard this law?

Simply this: that just at the point where the law begins to operate so beneficently and mercifully, I and others have seen the incision made, the canula inserted, the "life-giving" (?) air rapidly and freely inhaled, and the sufferer thereby brought back again to consciousness of his pain and nearly approaching end. He exists a few hours longer in agonizing anticipation of a certain and awful death, because robbed of the means by which it was intended his dissolution should be made painless and unconscious.

What, then, is gained? All agree that there is nothing in the operation itself of a curative nature, and in a case like this remedies have had full chance to do all in their power. The strength and vitality of the patient are already exhausted, and death is certain. Yet there are physicians and surgeons who, in a case such as described, believe they have not done their duty until they have so operated and brought the patient back to pain and consciousness. It is the customary thing to do, and therefore right. Nature's effort to secure a painless death is disregarded and defeated. "But," said one of these physicians to me a few days since, when discussing this matter, "after the operation, if there is such pain and suffering, *we must give them Morphia*"!

In other words, two wrongs will make a right.

I would not be understood to hold that tracheotomy is never justifiable, but only that it is very often unnecessarily performed and with such cruel results.

For the purpose of gaining valuable time, as to remove a foreign body from the throat, or even in a case of croup or diphtheria, which has rapidly approached asphyxiation without proper homœopathic treatment, *only when the means for applying such treatment are at last at hand*, it would be proper to resort to it. But when such means have been faithfully applied and failed, or when such means are not at hand, and the only result accomplished is to gain time to die in, with increase of suffering, such an operation is cruel and unnecessary, and opposed to the Divine ordering of things, which is for our highest good.

Nature's laws and nature's God are not cruel. Their purposes are accomplished with the least expenditure of force and the least suffering.

The law of "the least quantity of action," discovered by the great philosopher and scientist, Maupertisu, holds true in this, as in all other spheres.

